# White Cord Syndrome Following Anterior Cervical Discectomy and Fusion: A Case of Transient Quadriplegia With Complete Neurological Recovery

**DOI:** 10.7759/cureus.81961

**Published:** 2025-04-09

**Authors:** Felix Rivera Troia, Jose C Pérez López

**Affiliations:** 1 Orthopaedic Surgery, Ponce Health Sciences University, Ponce, PRI

**Keywords:** acute quadriplegia, anterior cervical discectomy and fusion (acdf), reperfusion injury, spine surgery, white cord syndrome

## Abstract

Cervical myelopathy is a progressive degenerative condition characterized by spinal cord compression, which often requires surgery to avoid further stepwise deterioration of this pathology. While anterior cervical discectomy and fusion (ACDF) is a well-established treatment, postoperative neurological deficits, although rare, remain a significant concern. White cord syndrome (WCS) is an uncommon cause of acute neurological deficit following spine surgery that is attributed to a reperfusion injury following decompression of a chronically impinged spinal cord. We present the case of a 47-year-old male with a preoperative diagnosis of cervical myelopathy (Nurick 4) who developed acute quadriplegia following ACDF. Initial imaging ruled out common postoperative complications, such as hematoma or hardware malposition, and an MRI revealed hyperintense signals consistent with WCS. Supportive management led to partial neurological recovery by postoperative day 3, followed by complete neurological recovery and marked functional and strength improvement beyond baseline by the five-month follow-up. This report aims to present a case of WCS, a rare complication following spinal decompression surgery, highlighting its diagnosis, management, and outcome.

## Introduction

Cervical myelopathy is a common degenerative condition of the cervical spinal cord, characterized by neurological dysfunction resulting from cord compression, most often at the C5 to C7 levels [1]. Symptoms are typically progressive and can range from neck pain and focal neurological deficits to less common presentations, such as headaches and muscle cramps [2]. The treatment of this condition is based on the severity of symptoms. While conservative management with anti-inflammatory medication and intermittent cervical immobilization is employed for mild cases, surgical intervention is often required for severe presentations of the disease [3,4].

Acute onset neurological deficit is among the most concerning and feared complications following spinal surgery. While studies suggest that this condition is rare, when it does occur, it most commonly affects the thoracic spine, among other locations [5]. Several common causes have been identified, including postoperative fluid collection, iatrogenic injury resulting from instrumentation, and trauma associated with decompression procedures [6]. However, in cases where no clear etiology can be determined, it is possible that acute postoperative neurological deficits may be attributed to reperfusion injury or white cord syndrome (WCS).

WCS is thought to be caused by reperfusion to an already chronically compressed and ischemic area of the spinal cord [7]. It is characterized by sudden neurological deficits in the acute postoperative period; however, cases of delayed symptom presentation have been reported [8,9]. We present a case of a patient who developed acute complete quadriplegia following anterior cervical discectomy and fusion (ACDF) for cervical myelopathy, ultimately diagnosed with WCS. By postoperative day 3, the patient had regained partial limb function and, by the five-month follow-up, demonstrated complete neurological recovery and remarkable functional and strength improvement compared to baseline.

## Case presentation

We present the case of a 47-year-old male with a history of hypertension and prediabetes, who was referred to the orthopedic spine clinic for cervical and lower back pain, accompanied with right upper extremity and right lower extremity weakness, paresthesias and lack of balance for the past three years. The patient described the symptoms as spontaneous in onset but progressively worsening. He rated the pain as 10/10 and denied any bowel or bladder dysfunction. His symptoms were initially aggravated by prolonged ambulation and relieved by rest or sitting but had progressed to occurring even at rest, particularly at night.

On physical examination, the patient stood well-balanced in both the coronal and sagittal plane but ambulated with an unsteady, wide-based gait and the assistance of a one-point cane. No tenderness or step-offs were noted on palpation of the cervical, thoracic, or lumbar spine. Muscle strength and sensation were impaired on both the lower and upper extremities, with more pronounced weakness on the right side (3.5/5). Deep tendon reflexes were hyper-reflexive and symmetric. Special tests such as the Spurling maneuver and finger escape tests were negative. However, a positive Hoffman sign was present, and bilateral feet clonus were noted.

**Figure 1:**
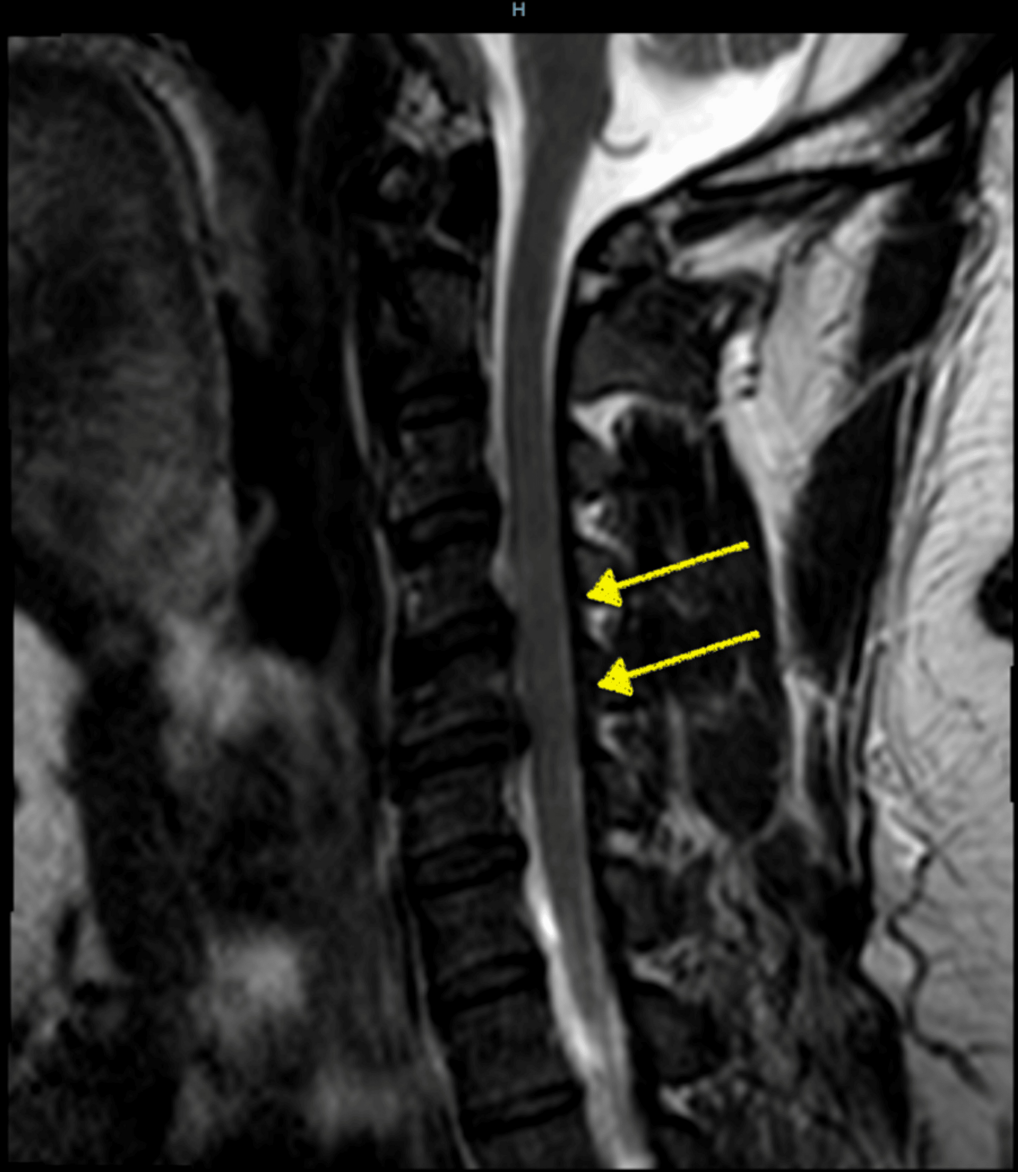
Sagittal view of an MRI of the cervical spine demonstrating stenosis of the spinal canal at C4 through C6 levels (yellow arrows) with absent CSF reserve.

The patient brought his magnetic resonance imaging (MRI) study of the cervical spine that showed prominent degenerative changes with severe spinal canal stenosis at C4 through C6 levels with absent CSF reserve and spinal cord signal changes (Figure 1). At this stage, the patient reported having failed months of conservative management, including physical therapy, chiropractic adjustments, and anti-inflammatory medication, and continued to experience impaired activities of daily living and reduced quality of life. Therefore, the risks and benefits of surgical intervention were discussed with the patient and his family, and an ACDF of C4 to C6 was planned in order to stop the stepwise progression of myelopathy and avoid further irreversible nerve damage.

ACDF was successfully performed with no intraoperative complications, and neurological monitoring was stable throughout the case. However, upon waking in the recovery room, the patient began to complain of not being able to move his upper or lower extremities. The surgeon was urgently called for an assessment of the patient. Initial postoperative examination revealed a complete loss of muscle strength (0/5) in the upper and lower extremities symmetrically and bilaterally. Sensation was also absent in all four limbs; however, hyperreflexia and clonus were noted, and the patient was transferred to ICU level care.

**Figure 2:**
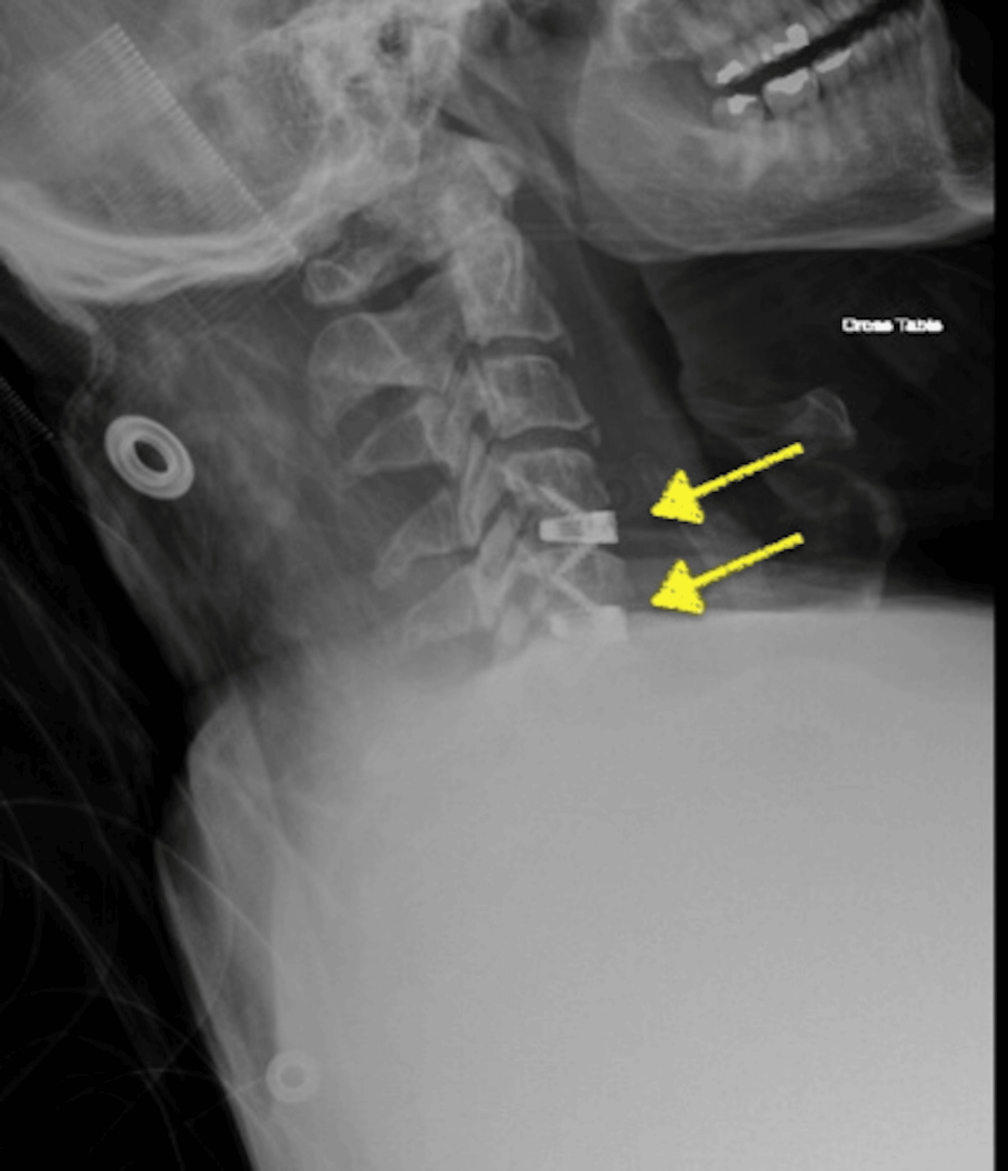
X-ray with lateral view of the cervical spine confirming postoperative proper screw placement and inter body spacers (yellow arrows) at C4-C6 levels.

**Figure 3:**
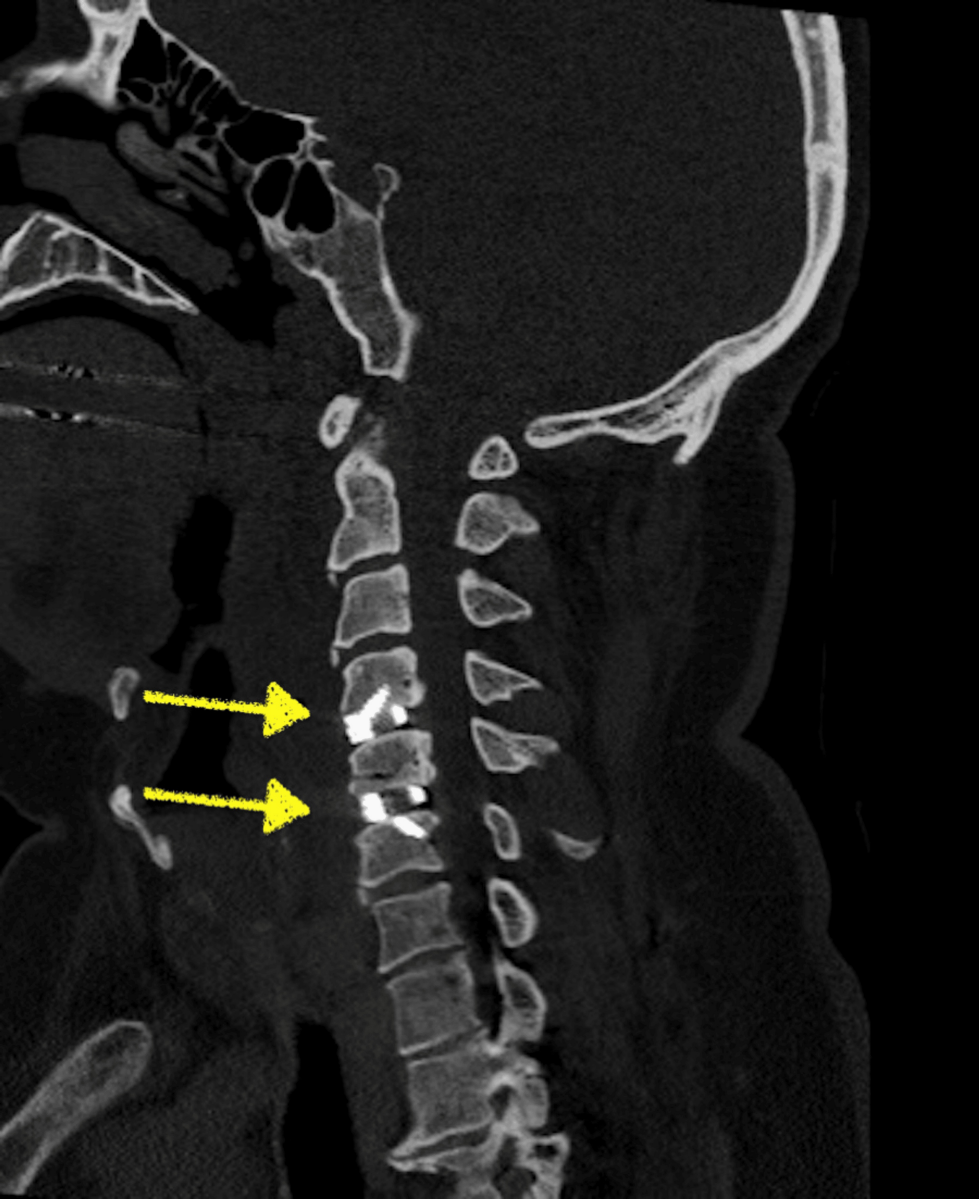
Sagittal view of non-contrast enhanced CT scan of the cervical spine further demonstrating proper screw placement (yellow arrows) at C4-C6 levels and absence of a hematoma formation.

**Figure 4:**
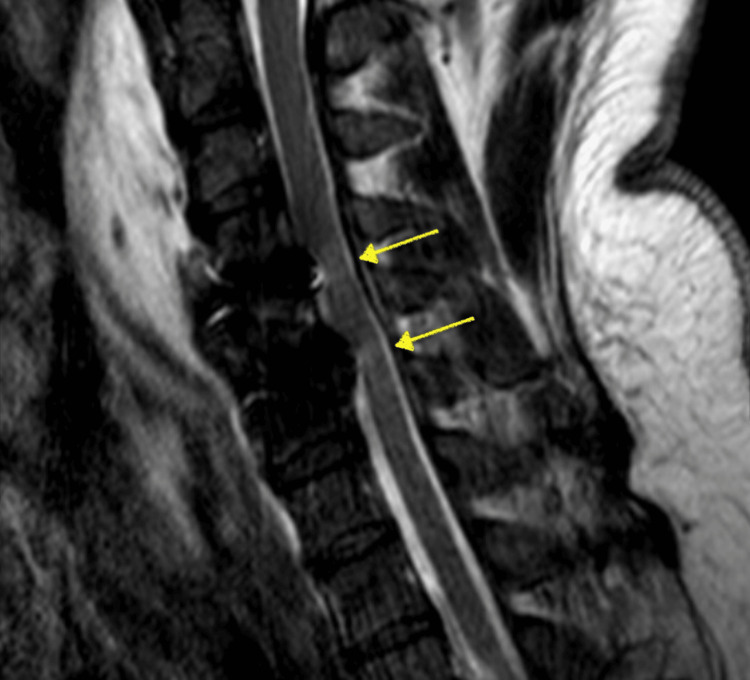
Sagittal view of a T2-weighted MRI of the cervical spine showing a hyperintense signal at the C4-C6 levels (yellow arrows).

The initial diagnosis of the surgeon was the possibility of an expanding hematoma causing cord compression or iatrogenic injury during instrumentation. Furthermore, a postoperative X-ray and computed tomography (CT) scan were performed to evaluate the spinal cord. X-ray demonstrated proper screw placement and inter-body spacer at the C4-C6 levels (Figure 2). The results of the CT scan were unremarkable (Figure 3), showing no sign of epidural hematoma formation or inappropriate screw placement. Subsequently, an MRI was ordered to rule out the possibility of a reperfusion injury. The results of this study demonstrated a hyperintense signal at the C4-C6 levels (Figure 4), correlating clinically with the findings observed in the patient and confirming the diagnosis of WCS. Therefore, the surgeon decided to start the patient on high-dose steroids and continued to monitor him for signs of improvement. 

Over the following days, he began to show signs of improvement. The patient began to feel pain over his body and started to regain muscle strength, starting distally and progressing proximally. By postoperative day 3, the patient had regained partial control and strength (3/5 of all 4 limbs) and was able to ambulate with a four-point walker. At this point, he was transferred to an inpatient care facility to further assist his rehabilitation. The patient was followed up in the clinic at two weeks and five months postoperatively. At five months postoperatively, the patient reported an overall satisfactory outcome with the procedure, along with no specific complaints, complete neurological recovery, and marked improvement of strength (4.5/5 in all four extremities) compared to the presurgical baseline (Table 1).

**Table 1:** Showing individual limb strengths from baseline through the five-month follow-up at different time points.

Limb	Preoperative (baseline)	Immediate postoperative period	Postoperative day #3	Five-month follow-up
Right upper extremity	3.5/5	0/5	3/5	4.5/5
Right lower extremity	3.5/5	0/5	3/5	4.5/5
Left upper extremity	4/5	0/5	4/5	4.5/5
Left lower extremity	4/5	0/5	4/5	4.5/5

## Discussion

Acute neurological deficits are an uncommon, well-established complication following spinal surgery. The degree of injury falls along a spectrum from transient paresthesia to permanent quadriplegia [10]. Barrett et al. suggest that the most common cause of postoperative neurological deficit is post-surgery fluid collection, followed by improper position of instruments and traumatic decompressions [6]. WCS is a rare cause of postoperative acute neurological deficit following spinal decompression surgery, which leads to neurological deterioration despite the absence of any identifiable perioperative injury [11]. First described by Chin et al. in 2013, WCS is thought to occur due to spinal cord injury from rapid expansion and reperfusion, triggering free radical release and oxidative stress [12].

MRI is the preferred method for diagnosis of WCS, characterized by the presence of a hyperintense signal in the spinal cord on T2-weighted MRI [13]. Liao et al. described diagnostic criteria for characterizing these injuries that include severe spinal cord compression, paralysis that occurs within three hours following surgical intervention, progressively ascending motor and sensory dysfunction, and the absence of other identifiable causes [14]. Our patient exhibited at least three of these criteria.

Furthermore, WCS can be managed surgically or conservatively. While some cases have been managed with secondary surgical interventions [15], others have found comparable results with conservative treatment methods, including the use of neuropathic pain medication, steroids, and physical therapy [7,8,11,16]. However, most of these conservatively managed cases showed slow and progressive improvement [16,17], unlike our case that showed rapid recovery, with enough strength regained (3/5) to ambulate with a four-point walker in just three days.

Few cases have been reported in the literature, and of those reported, most required subsequent procedures to correct the neurological manifestation [9,12,15,18]. Zhang et al. reported three cases of WCS following anterior cervical corpectomy and fusion [18]. While one patient required additional surgical intervention, the other two experienced resolution of neurological deficits with conservative management alone. Similarly, Giammalva et al. described a case of WCS after a double-level ACDF, in which the patient developed postoperative tetraplegia, comparable to our case [19]. Their patient, like ours, was managed conservatively and showed partial recovery by postoperative day 3, further supporting the conservative approach taken in our case.

## Conclusions

WCS is a rare cause of acute neurological deficit following spine surgery, caused by reperfusion of a chronically compressed and ischemic spinal cord. Management should focus on ruling out common causes of neurologic injury through a thorough workup and physical exam. We present the case of a patient managed conservatively who regained enough neurological function and strength to ambulate with a four-point walker by postoperative day 3 and showed complete neurological recovery and marked functional and strength improvement from baseline at the five-month follow-up. This case highlights the diagnosis, management, and outcome of an uncommon complication of spine surgery.
